# Dysfunction of Wnt signaling and synaptic disassembly in neurodegenerative diseases

**DOI:** 10.1093/jmcb/mjt049

**Published:** 2014-02

**Authors:** Silvia A. Purro, Soledad Galli, Patricia C. Salinas

**Affiliations:** 1 Department of Neurodegenerative Disease, UCL Institute of Neurology, Queen Square, London WC1N 3BG, UK; 2 Department of Cell and Developmental Biology, University College London, London WC1E 6BT, UK

**Keywords:** Wnt signaling, synaptic disassembly, degenerative diseases, Alzheimer's disease, Dkk1, synaptic maintenance

## Abstract

The molecular mechanisms that regulate synapse formation have been well documented. However, little is known about the factors that modulate synaptic stability. Synapse loss is an early and invariant feature of neurodegenerative diseases including Alzheimer's (AD) and Parkinson's disease. Notably, in AD the extent of synapse loss correlates with the severity of the disease. Hence, understanding the molecular mechanisms that underlie synaptic maintenance is crucial to reveal potential targets that will allow the development of therapies to protect synapses. Wnts play a central role in the formation and function of neuronal circuits. Moreover, Wnt signaling components are expressed in the adult brain suggesting their role in synaptic maintenance in the adult. Indeed, blockade of Wnts with the Wnt antagonist Dickkopf-1 (Dkk1) causes synapse disassembly in mature hippocampal cells. Dkk1 is elevated in brain biopsies from AD patients and animal models. Consistent with these findings, Amyloid-β (Aβ) oligomers induce the rapid expression of Dkk1. Importantly, Dkk1 neutralizing antibodies protect synapses against Aβ toxicity, indicating that Dkk1 is required for Aβ-mediated synapse loss. In this review, we discuss the role of Wnt signaling in synapse maintenance in the adult brain, particularly in relation to synaptic loss in neurodegenerative diseases.

## Introduction

Appropriate formation, maintenance, and elimination of synapses are crucial to guarantee the formation of a functional nervous system. In the last decade, great advances have been made in understanding the molecular mechanisms that regulate the formation and function of synapses. However, the factors that modulate synaptic maintenance in the adult brain are less understood. Several neurodegenerative diseases such as Alzheimer's (AD) and Parkinson's disease exhibit early loss of synapses, which correlates with the manifestation of cognitive and/or motor symptoms and subsequent neuronal degeneration. Therefore, protecting synapses early in the disease could provide an effective therapeutic approach for the treatment of these diseases.

**Figure 1:**
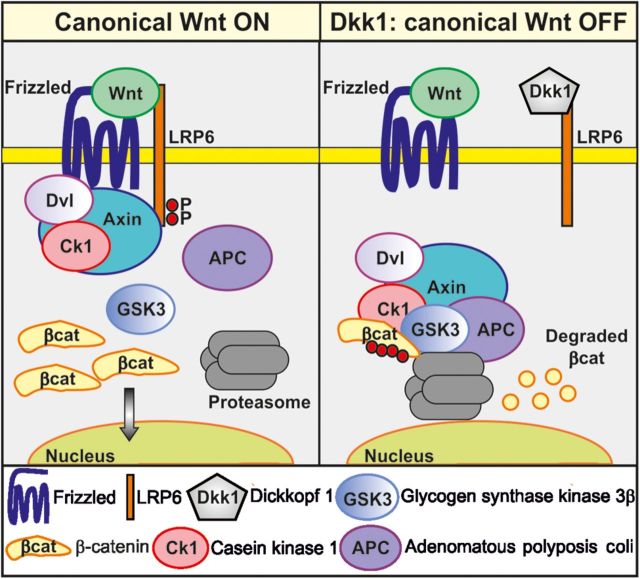
Canonical Wnt signaling. Left panel: binding of Wnts to Frizzled receptors and co-receptor LRP5/6 induces association of Axin with phosphorylated LRP6 and recruitment of Dvl and CK1 in a complex that binds and inhibits Gsk3β. Blockade of Gsk3β results in the accumulation of β-catenin in the cytoplasm, which translocates to the nucleus to induce gene expression. Right panel: Dkk1 is a secreted Wnt antagonist that binds to the LRP5/6. Hence, Wnts can no longer signal through the canonical pathway and inhibit Gsk3β, which in turn phosphorylates β-catenin, targeting it for degradation by the proteasome.

Wnts are a large family of secreted glycoproteins that promote synaptogenesis and regulate synaptic function in both vertebrates and invertebrates (Inestrosa and Arenas, 2010; Shen and Cowan, 2010; Budnik and Salinas, 2011; Salinas, 2012). Binding of Wnts to Frizzled (Fz) receptors can activate different pathways. In the canonical or β-catenin pathway, Wnts act through Fz receptors and the co-receptor low density lipoprotein receptor-related protein 5/6 (LRP5 or LRP6), requiring the scaffold protein Dishevelled (Dvl; Figure 1). Activation of this pathway results in the inhibition of the serine/threonine kinase glycogen synthase kinase-3 (Gsk3), which in the absence of Wnts phosphorylates β-catenin increasing its instability. In the presence of Wnts, β-catenin is stabilized and translocates to the nucleus where it activates the transcription of target genes (Clevers and Nusse, 2012). A divergent canonical pathway, independent of transcription, requires the plus-end microtubule binding protein Adenomatous polyposis coli to regulate axonal remodeling (Ciani et al*.*, 2004; Purro et al*.*, 2008), a process that precedes presynaptic assembly. The non-canonical pathways include the planar cell polarity pathway, with the activation of Rho GTPases and Jun N-terminal kinase (JNK), and the calcium pathway, where an increase in intracellular calcium activates the calcium/calmodulin-dependent protein kinase II (CaMKII) and protein kinase C. Wnts recruit synaptic proteins to the presynaptic terminals through the activation of the canonical pathway (Hall et al*.*, 2000; Packard et al*.*, 2002; Ahmad-Annuar et al*.*, 2006; Davis et al*.*, 2008) and also recruit postsynaptic proteins by either the canonical or non-canonical pathway (Farias et al*.*, 2009; Ciani et al*.*, 2011). Therefore, Wnts can act through different signaling cascades to function as synaptogenic molecules.

The role of Wnts in synapse formation, growth, and plasticity has been well documented both in the central and peripheral nervous systems (Inestrosa and Arenas, 2010; Budnik and Salinas, 2011; Koles and Budnik, 2012; Salinas, 2012). However, Wnts, their receptors, and many of the components of the signaling pathway are also expressed in the adult brain, suggesting that they might have a role in synaptic stability. Here we review recent studies that strongly suggest a role of Wnt signaling in the maintenance of synapses in the adult brain and indicate that dysfunction of Wnt signaling may contribute to the loss of synapses in AD. We will also discuss how modulation of Wnt signaling could be used as a therapeutic strategy for the treatment of neurodegenerative diseases beyond AD.

## Wnt signaling on synaptic formation and function

A variety of evidence demonstrates a role for Wnts in the formation and growth of synapses. In the cerebellum, Wnt7a is required for the formation of synapses between granule cell dendrites and mossy fiber axons by promoting axonal remodeling and the recruitment of presynaptic proteins (Hall et al*.*, 2000). Prior to the formation of synaptic boutons, Wnts induce extensive remodeling of axons that results from a decrease in axon extension and the concomitant increase of the size of their growth cones (Hall et al*.*, 2000; Purro et al*.*, 2008). Gain and loss of function studies demonstrate that Wnt7a and Dvl1 are crucial for the recruitment of presynaptic proteins to future synaptic boutons, therefore, increasing the number and size of synaptic vesicle recycling sites (Ahmad-Annuar et al*.*, 2006). Consistent with a role of Wnts in synapse formation, the *Wnt7a/Dvl1* double mutant mice exhibit defects in the frequency, but not amplitude, of miniature excitatory postsynaptic currents (Ahmad-Annuar et al*.*, 2006). Further studies demonstrate that downstream of Dvl1 lays Gsk3, as inhibition of this kinase mimics the effect of Wnt7a (Hall et al*.*, 2000). In cultured neurons, Wnt5a also induces the recruitment of presynaptic components, but the mechanism remains elusive (Paganoni et al*.*, 2010; Varela-Nallar et al*.*, 2012). Together, these findings demonstrate that several Wnts can regulate the formation of synapses by promoting the assembly of the presynaptic terminal.

Wnts can signal through many receptors to induce synapse formation. For example, both Wnt3a via Fz1 and Wnt5a via Ror tyrosine receptors increase the number of presynaptic sites on hippocampal cultures (Varela-Nallar et al*.*, 2009; Paganoni et al*.*, 2010). The receptor Fz5 is required for Wnt7a to stimulate synaptogenesis in the hippocampus, as shRNA knockdown of Fz5 blocks Wnt7a function (Sahores et al*.*, 2010). Apart from these findings, little is known about the receptors needed for other synaptogenic Wnts and their different targets. Interestingly, endogenous Wnts are required for surface localization of Fz5 and for the formation of synaptic sites elicited by neuronal activity (Sahores et al*.*, 2010). These findings revealed an important role for Wnts in activity-mediated changes at the synapse. However, further studies are required to elucidate the mechanisms that control the trafficking of Wnt receptors and their consequences on synapse formation and growth.

Wnts also promote postsynaptic assembly with the added complexity that different Wnts might modulate either side of the synapse through different pathways. In hippocampal neurons, Wnt7a signals bidirectionally to the synapse. Wnt7a increases the number and function of excitatory synapses by activating canonical Wnt signaling at the presynapse (Cerpa et al*.*, 2008; Ciani et al*.*, 2011). Signaling directly to the postsynaptic side, Wnt7a induces the localization and activation of CaMKII at spines, increasing synaptic strength. Indeed, *Wnt7a-Dvl1*-deficient mice exhibit defects in spine morphogenesis and excitatory synaptic transmission in the hippocampus (Ciani et al*.*, 2011). Another complexity lies in the fact that different Wnts modulate distinct types of synapses. Wnt7a specifically regulates the formation of excitatory synapses without changing the number of inhibitory ones (Ciani et al*.*, 2011), while Wnt5a promotes synaptic assembly on both excitatory and inhibitory hippocampal neurons (Cuitino et al*.*, 2010; Varela-Nallar et al*.*, 2010). In contrast to Wnt7a, Wnt5a induces postsynaptic assembly and increases the levels of γ-aminobutyric acid type a (GABA_A_) receptors by activating non-canonical Wnt signaling in hippocampal neurons (Farias et al*.*, 2009; Cuitino et al*.*, 2010; Varela-Nallar et al*.*, 2010). Wnt5a increases the amplitude but not the frequency of miniature inhibitory postsynaptic currents, suggesting that Wnt5a increases and maintains GABA receptors on the postsynaptic membrane without affecting GABA release from the presynaptic side (Cuitino et al*.*, 2010). Interestingly, Wnt5a activates different Wnt pathways at the postsynapse. The recruitment of postsynaptic proteins is achieved by activation of JNK, whereas the modulation of GABA receptors requires CaMKII (Farias et al*.*, 2009; Cuitino et al*.*, 2010; Varela-Nallar et al*.*, 2010). *In vivo* data on Wnt5a function and *in vitro* detailed studies are needed to clarify the mechanism and targets on each side of the synapse. Future experiments will elucidate the mechanisms by which different Wnts regulate the formation of different types of synapses, and whether different mechanisms govern the assembly of the pre- and postsynaptic sites.

## Modulation of synaptic maintenance by Wnt signaling

The expression of Wnts and signaling components in the adult brain suggests their potential role in synaptic maintenance (Shimogori et al*.*, 2004; Gogolla et al*.*, 2009; Sahores et al*.*, 2010; Seib et al*.*, 2013). Indeed, experiments using a secreted Wnt antagonist, Dickkopf-1 (Dkk1), demonstrate a role for Wnts in synaptic maintenance in mature hippocampal neurons. Dkk1 binds to LRP6 inhibiting Wnt canonical signaling (Niehrs, 2006). In mature neurons, short-term exposure to Dkk1 induces a decrease in the number and size of synaptic protein clusters, decreases the number of functional synaptic vesicle recycling sites, and promotes dispersion of pre- and postsynaptic proteins indicating loss of synapses. This effect was achieved by a redistribution of the synaptic proteins, as the total level of proteins remained unaffected (Purro et al*.*, 2012). Ultrastructural studies showed that Dkk1 decreases the length of the active zone and the postsynaptic density area of remaining synapses, consistent with the view that remaining synapses are smaller or have shrunk. Indeed, time-lapse recordings of VAMP-mRFP-labeled stable synaptic sites indicate that Wnt blockade by Dkk1 rapidly disassembles synapses, decreasing the number, size, and intensity of synaptic puncta (Purro et al*.*, 2012). Given that the effect of Dkk1 can be reverted by Wnts or inhibition of Gsk3 (unpublished data), these findings strongly suggest that endogenous Wnts expressed by mature neurons are crucial for the maintenance of synaptic connections in the adult brain.

## AD and Wnt signaling

It is estimated that 35 million people suffer dementia worldwide and the number of cases is expected to triple by 2050 (Prince and Jackson, 2009) having a significant economical and social impact. AD is the most common form of dementia with increasing costs on health systems and society. However, existing treatments are only palliatives and no cure for this disease has yet been found. Memory loss, changes in personality and behavior, and a gradual loss of mental abilities are the most characteristic symptoms of the disease (Prince and Jackson, 2009).

In the brain, tangles and plaques, the hallmarks of AD, accumulate and cause neurodegeneration (Selkoe, 2011). Neurofibrillary tangles (NFTs) are aggregates of hyperphosphorylated Tau, a microtubule binding protein, while plaques are mainly deposits of amyloid-β peptides (Aβ), the product from the shedding of amyloid-β precursor protein (APP) (Selkoe et al*.*, 1996). Although plaques and NFTs are toxic, synapses are lost before plaque deposition and NFTs are significant in the brain. It is widely accepted that soluble Aβ aggregates cause synapse dysfunction and loss, probably linked to the cognitive impairments characteristic of AD (Ferreira and Klein, 2011). During the disease, there is a significant loss of synapses, which correlates best with the cognitive decline (Terry et al*.*, 1991; Palop and Mucke, 2010). Evidence from *in vitro* and *in vivo* studies indicates that soluble Aβ aggregates like dimers, trimers, and different sized oligomers are the synaptotoxic species (Walsh and Selkoe, 2007; Ferreira and Klein, 2011; Larson and Lesne, 2012). However, the mechanisms by which Aβ induces synaptic damage are poorly understood.

The toxic effects of Aβ on synapses could be due to its impact on the expression or function of synaptic maintenance factors and suggest that protection of synapses early in the disease could ameliorate the impact of Aβ toxicity. Therefore, understanding the molecular mechanisms that regulate synaptic maintenance is crucial for developing approaches to block the effect of Aβ and therefore protect or even restore neuronal connectivity.

Several studies link a deficiency in canonical Wnt signaling with AD (see review in (Inestrosa et al*.*, 2012)). Dkk3, highly related to Dkk1, is elevated in plasma and cerebrum spinal fluid from AD patients, supporting that deregulation of Wnt signaling takes place during AD (Zenzmaier et al*.*, 2009). Further evidence comes from the finding that Aβ binds to Fz5, although the significance of this finding remains to be explored (Magdesian et al*.*, 2008). The Wnt antagonist Dkk1 is elevated in post-mortem brain samples from AD patients and brains from transgenic AD animal models (Caricasole et al*.*, 2004; Rosi et al*.*, 2010), suggesting that dysfunction of Wnt pathway could contribute to the loss of synapses in AD. Importantly, genome-wide association studies showed a link between a variant of the LRP6 receptor with late onset of AD. This identified LRP6 variant exhibits lower activation of gene transcription upon addition of Wnt3a in heterologous cells (De Ferrari et al*.*, 2007). Together, these studies strongly suggest that dysfunction of the canonical Wnt pathway is associated with AD and raise the interesting possibility that Dkk1 mediates the loss of synapses characteristic of AD.

**Figure 2:**
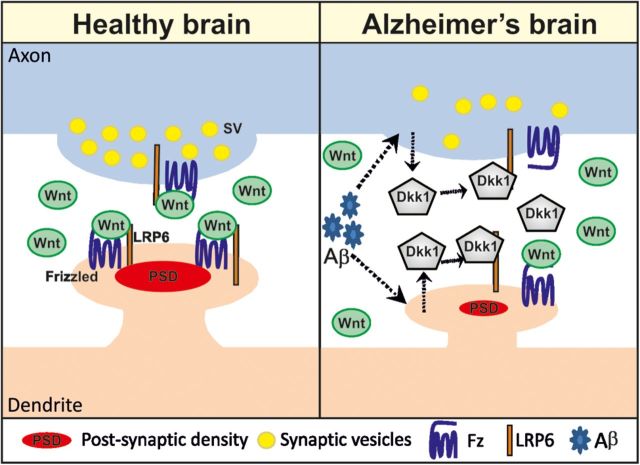
Dkk1 is crucial for synaptic disassembly and loss induced by Aβ. Left panel: Wnts by binding to Frizzled and LRP5/6 regulate the maintenance of synapses at the pre- and postsynaptic site in the adult brain. Right panel: Aβ induces Dkk1 expression (dotted arrows). Dkk1 blocks canonical Wnt signaling, activating a dispersal mechanism of synaptic components leading to decrease in the size of the synapse and disassembly.

How does Aβ affect Wnt signaling at the synapse? Studies on mouse brain slices have demonstrated that short-term exposure to Aβ induces the loss of synapses concomitantly with increased expression of Dkk1 (Purro et al*.*, 2012) (Figure 2). Importantly, a neutralizing antibody against Dkk1 protected synapses from the deleterious effect of Aβ. As mentioned above, Wnt blockade by Dkk1 quickly disassembles synaptic sites resulting in the loss of synapses. We propose that the effect of Dkk1 is due to blockade of endogenous Wnt ligands that are crucial for synapse maintenance. Several results support this conclusion. First, several Wnt factors, their receptors, and components of the Wnt signaling pathway are expressed in the adult brain. Second, the synaptic effect of Dkk1 can be blocked by Wnt ligands. Third, activation of the canonical Wnt pathway by inhibiting Gsk3 blocks the effect of Dkk1 (unpublished data). Together, these results demonstrate that endogenous Wnts contribute to synaptic stability and this crucial function is disrupted by the accumulation of Aβ, which results in the elevation of Dkk1, otherwise not present in the healthy brain. Dkk1 in turn induces the loss of synapses. These findings strongly suggest a link between dysfunction of Wnt signaling and Aβ-mediated synaptotoxicity.

## Dysfunction of Wnt signaling, cognitive decline and synaptic disassembly

**Figure 3:**
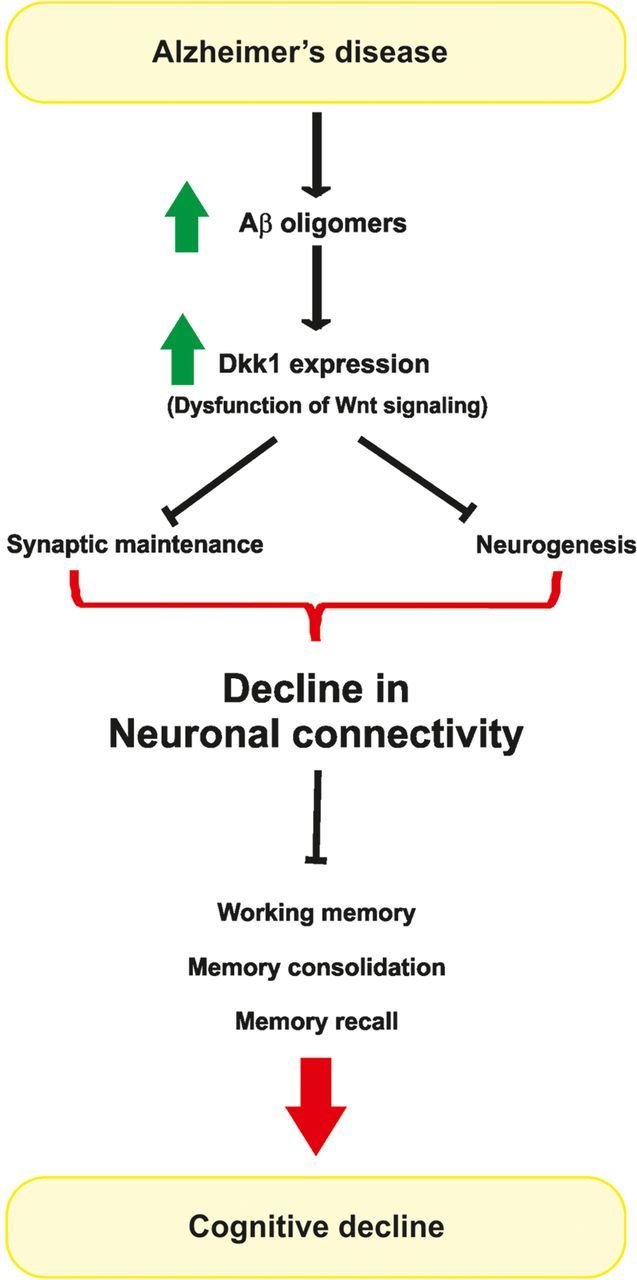
Potential stages in the synaptic pathogenesis in AD. During the disease, accumulation of Aβ oligomers induces the expression of the Wnt antagonist Dkk1. Dkk1 induces synaptic disassembly and inhibits neurogenesis, therefore resulting in a decrease in global synaptic connectivity in the brain. Decline in synaptic connectivity would contribute to impairment in memory, suggesting that Aβ/Dkk1-mediated loss of synapses underlies the cognitive decline observed at early stages of AD.

Loss of synapses, spines, and dendrites precedes neuronal degeneration and is thought to underlie the symptoms in several neurodegenerative diseases (Lin et al*.*, 2009; Shankar and Walsh, 2009; Milnerwood and Raymond, 2010; Moreno and Mallucci, 2010). In AD, there is a strong correlation between synaptic loss and cognitive decline, and this occurs independently of neuronal death (Naslund et al*.*, 2000; Shankar and Walsh, 2009). A recent study showed that Dkk1 expression increases with age in the mouse hippocampus concomitantly with a decline in spatial working memory (Seib et al*.*, 2013). Importantly, loss of Dkk1 expression using ShRNA in the hippocampus of aged mice enhances spatial working memory and memory consolidation (Seib et al*.*, 2013). These data indicate that dysfunction of Wnt signaling causes cognitive impairment. Interestingly, Dkk1 and the secreted frizzled-related protein 3 (Sfrp3), both antagonists of Wnts, affect the stem cell niche in the adult dentate gyrus (Jang et al*.*, 2013; Seib et al*.*, 2013). However, the effect of these Wnt inhibitors on synapses and connectivity remains to be studied. Given the effect of Dkk1 on the rapid disassembly of synapses (Purro et al*.*, 2012), we would predict that knockdown of Dkk1 could induce changes in synaptic connectivity by directly affecting existing neurons and synapses. At any rate, these findings demonstrate the importance of endogenous Wnt signaling in hippocampal-mediated behavior. They also suggest that Wnt dysfunction could contribute not only to age-related cognitive decline but also to render synapses more vulnerable to toxic factors like Aβ oligomers (Figure 3).

The expression of Wnt proteins changes with age. For example, Wnt7a/b levels increase between 6 and 12 months of age in mouse hippocampus, and this is followed by a decrease in the number of synapses (Gogolla et al*.*, 2009). This raises the interesting possibility that Wnt-dependent synaptic stability decreases with age. In a recent study, it was shown that prolonged exposure to enriched environment (EE) enlarges dendritic spines, enhances hippocampal plasticity, and alleviates the synaptotoxicity of Aβ oligomers from AD brain patients (Li et al*.*, 2013). This highlights the importance of synaptic stability and function to overcome the toxic effects of Aβ. It is well documented that EE produces a robust increase in the number of excitatory synapses in the CNS (Moser et al*.*, 1997; Gogolla et al*.*, 2009; Baroncelli et al*.*, 2010; Bednarek and Caroni, 2011) and interestingly, EE also increases the level of Wnt7a/b in CA3 pyramidal neurons in the hippocampus (Gogolla et al*.*, 2009). Indeed, blockade of Wnt signaling with Sfrp1 suppresses the effect of EE on synaptic remodeling (Gogolla et al*.*, 2009). As the levels of Wnts and the number of synapses decrease in aged mice, we propose that remaining synapses might become more vulnerable to Aβ toxicity and therefore to disassembly in the aging brain. In contrast, increasing the number of synapses, by exposure to sensory, motor, or social stimuli might be protective during the early stages of the disease. Interestingly, restoration of Wnt signaling in aged individuals may provide an alternative therapy to prevent the disassembly of synapses and concomitant symptoms of AD.

Aβ is known to increase the expression of the Wnt antagonist Dkk1 (Caricasole et al*.*, 2004; Killick et al*.*, 2014; Purro et al*.*, 2012), whereas blockade of Dkk1 suppresses Aβ synaptotoxicity (Purro et al*.*, 2012). However, the mechanism by which Aβ and Dkk1 induce synaptic disassembly still remains elusive. Recently, it was shown that Aβ induces intracellular accumulation of the survival factor clusterin and indeed, knockdown of clusterin in primary neurons reduced Aβ toxicity and the upregulation of Dkk1 (Killick et al*.*, 2014), suggesting that Dkk1-mediated Aβ neurotoxicity occurs via clusterin. Interestingly, clusterin accumulation would induce activation of p53 pathway and certainly, inhibition of p53 blocks Aβ induction of Dkk1 (Killick et al*.*, 2014). These studies provide for the first time a mechanism that links dysfunctional Wnt signaling with Aβ toxicity. It would be crucial to establish whether *in vivo* p53-mediated increase of Dkk1 also contributes to synaptic disassembly induced by Aβ.

## Concluding remarks

Wnt signaling is critical for the formation of neuronal circuits by modulating axon pathfinding, dendritic development, and synaptic assembly and function. Furthermore, Wnts mediate synaptic structural changes induced by neuronal activity or experience. The role of Wnt signaling in the regulation of synaptic maintenance in the adult brain is now beginning to emerge. Wnt signaling molecules are present in the adult brain. Indeed, certain Wnts change their expression throughout life and these changes are accompanied by changes in the number of synapses. Whether the decrease in synapses is indeed due to the lower levels of Wnts needs to be corroborated. Future studies should provide further understanding on the contribution of Wnts, their receptors, and downstream signaling components during aging. Importantly, *in vivo* studies will provide pivotal evidence for the contribution of Wnts to synaptic maintenance during aging.

In neurodegenerative diseases like AD, the loss of synapses that occurs at early stages correlates with cognitive decline, and these arise prior to the onset of neuronal degeneration. It is now clear that Wnt signaling plays a role in the onset of neurodegenerative diseases like AD. Certainly, dysfunction of Wnt pathway mediates Aβ-induced synaptic disassembly and might contribute to memory impairment and cognitive decline. Different components of the Wnt pathway like Dkk1, the Wnt antagonist Dkk3, and the co-receptor LRP6 are now linked to AD. Importantly, Dkk1 mediates the effect of Aβ on synaptic disassembly. Future studies will shed new light into the mechanisms underlying Wnt–Aβ-mediated synaptic disassembly and whether dysfunction in Wnt signaling also contributes to the loss of synapses in other neurodegenerative diseases.

Wnts' contribution to synaptic maintenance in the adult raises the interesting possibility that new therapies may arise by using Wnts or small molecules that mimic Wnt signaling to protect synapses. By protecting synapses at early stages of neurodegenerative diseases, these new therapies may ameliorate the symptoms and potentially delay the onset of neuronal degeneration.

## Funding

Our work is supported by the Medical Research Council (MRC), Wellcome Trust, Alzheimer's Research Trust, Parkinsons’ UK charity, and the European Union F7.

**Conflict of interest:** none declared.

## References

[MJT049C1] Ahmad-Annuar A., Ciani L., Simeonidis I. (2006). Signaling across the synapse: a role for Wnt and Dishevelled in presynaptic assembly and neurotransmitter release. J. Cell Biol..

[MJT049C2] Baroncelli L., Braschi C., Spolidoro M. (2010). Nurturing brain plasticity: impact of environmental enrichment. Cell Death Differ..

[MJT049C3] Bednarek E., Caroni P. (2011). β-adducin is required for stable assembly of new synapses and improved memory upon environmental enrichment. Neuron.

[MJT049C4] Budnik V., Salinas P.C. (2011). Wnt signaling during synaptic development and plasticity. Curr. Opin. Neurobiol..

[MJT049C5] Caricasole A., Copani A., Caraci F. (2004). Induction of Dickkopf-1, a negative modulator of the Wnt pathway, is associated with neuronal degeneration in Alzheimer's brain. J. Neurosci..

[MJT049C6] Cerpa W., Godoy J.A., Alfaro I. (2008). Wnt-7a modulates the synaptic vesicle cycle and synaptic transmission in hippocampal neurons. J. Biol. Chem..

[MJT049C7] Ciani L., Krylova O., Smalley M.J. (2004). A divergent canonical WNT-signaling pathway regulates microtubule dynamics: dishevelled signals locally to stabilize microtubules. J. Cell Biol..

[MJT049C8] Ciani L., Boyle K.A., Dickins E. (2011). Wnt7a signaling promotes dendritic spine growth and synaptic strength through Ca^2+^/Calmodulin-dependent protein kinase II. Proc. Natl Acad. Sci. USA.

[MJT049C9] Clevers H., Nusse R. (2012). Wnt/β-catenin signaling and disease. Cell.

[MJT049C10] Cuitino L., Godoy J.A., Farias G.G. (2010). Wnt-5a modulates recycling of functional GABAA receptors on hippocampal neurons. J. Neurosci..

[MJT049C11] Davis E.K., Zou Y., Ghosh A. (2008). Wnts acting through canonical and noncanonical signaling pathways exert opposite effects on hippocampal synapse formation. Neural Dev..

[MJT049C12] De Ferrari G.V., Papassotiropoulos A., Biechele T. (2007). Common genetic variation within the low-density lipoprotein receptor-related protein 6 and late-onset Alzheimer's disease. Proc. Natl Acad. Sci. USA.

[MJT049C13] Farias G.G., Alfaro I.E., Cerpa W. (2009). Wnt-5a/JNK signaling promotes the clustering of PSD-95 in hippocampal neurons. J. Biol. Chem..

[MJT049C14] Ferreira S.T., Klein W.L. (2011). The Aβ oligomer hypothesis for synapse failure and memory loss in Alzheimer's disease. Neurobiol. Learn. Mem..

[MJT049C15] Gogolla N., Galimberti I., Deguchi Y. (2009). Wnt signaling mediates experience-related regulation of synapse numbers and mossy fiber connectivities in the adult hippocampus. Neuron.

[MJT049C16] Hall A.C., Lucas F.R., Salinas P.C. (2000). Axonal remodeling and synaptic differentiation in the cerebellum is regulated by WNT-7a signaling. Cell.

[MJT049C17] Inestrosa N.C., Arenas E. (2010). Emerging roles of Wnts in the adult nervous system. Nat. Rev. Neurosci..

[MJT049C18] Inestrosa N.C., Montecinos-Oliva C., Fuenzalida M. (2012). Wnt signaling: role in Alzheimer disease and schizophrenia. J. Neuroimmune. Pharmacol..

[MJT049C19] Jang M.H., Bonaguidi M.A., Kitabatake Y. (2013). Secreted frizzled-related protein 3 regulates activity-dependent adult hippocampal neurogenesis. Cell Stem Cell.

[MJT049C20] Killick R., Ribe E.M., Al-Shawi R. (2014). Clusterin regulates β-amyloid toxicity via Dickkopf-1-driven induction of the wnt-PCP-JNK pathway. Mol. Psychiatry.

[MJT049C21] Koles K., Budnik V. (2012). Wnt signaling in neuromuscular junction development. Cold Spring Harb. Perspect. Biol..

[MJT049C22] Larson M.E., Lesne S.E. (2012). Soluble Aβ oligomer production and toxicity. J. Neurochem..

[MJT049C23] Li S., Jin M., Zhang D. (2013). Environmental novelty activates β2-adrenergic signaling to prevent the impairment of hippocampal LTP by Aβ oligomers. Neuron.

[MJT049C24] Lin L., Lesnick T.G., Maraganore D.M. (2009). Axon guidance and synaptic maintenance: preclinical markers for neurodegenerative disease and therapeutics. Trends Neurosci..

[MJT049C25] Magdesian M.H., Carvalho M.M., Mendes F.A. (2008). Amyloid-β binds to the extracellular cysteine-rich domain of Frizzled and inhibits Wnt/β-catenin signaling. J. Biol. Chem..

[MJT049C26] Milnerwood A.J., Raymond L.A. (2010). Early synaptic pathophysiology in neurodegeneration: insights from Huntington's disease. Trends Neurosci..

[MJT049C27] Moreno J.A., Mallucci G.R. (2010). Dysfunction and recovery of synapses in prion disease: implications for neurodegeneration. Biochem. Soc. Trans..

[MJT049C28] Moser M.B., Trommald M., Egeland T. (1997). Spatial training in a complex environment and isolation alter the spine distribution differently in rat CA1 pyramidal cells. J. Comp. Neurol..

[MJT049C29] Naslund J., Haroutunian V., Mohs R. (2000). Correlation between elevated levels of amyloid β-peptide in the brain and cognitive decline. JAMA.

[MJT049C30] Niehrs C. (2006). Function and biological roles of the Dickkopf family of Wnt modulators. Oncogene.

[MJT049C31] Packard M., Koo E.S., Gorczyca M. (2002). The Drosophila Wnt, wingless, provides an essential signal for pre- and postsynaptic differentiation. Cell.

[MJT049C32] Paganoni S., Bernstein J., Ferreira A. (2010). Ror1-Ror2 complexes modulate synapse formation in hippocampal neurons. Neuroscience.

[MJT049C33] Palop J.J., Mucke L. (2010). Amyloid-β-induced neuronal dysfunction in Alzheimer's disease: from synapses toward neural networks. Nat. Neurosci..

[MJT049C34] Prince M., Jackson J. (2009). World Alzheimer report 2009.

[MJT049C35] Purro S.A., Ciani L., Hoyos-Flight M. (2008). Wnt regulates axon behavior through changes in microtubule growth directionality: a new role for adenomatous polyposis coli. J. Neurosci..

[MJT049C36] Purro S.A., Dickins E.M., Salinas P.C. (2012). The secreted Wnt antagonist Dickkopf-1 is required for amyloid β-mediated synaptic loss. J. Neurosci..

[MJT049C37] Rosi M.C., Luccarini I., Grossi C. (2010). Increased Dickkopf-1 expression in transgenic mouse models of neurodegenerative disease. J. Neurochem..

[MJT049C38] Sahores M., Gibb A., Salinas P.C. (2010). Frizzled-5, a receptor for the synaptic organizer Wnt7a, regulates activity-mediated synaptogenesis. Development.

[MJT049C39] Salinas P.C. (2012). Wnt signaling in the vertebrate central nervous system: from axon guidance to synaptic function. Cold Spring Harb. Perspect. Biol..

[MJT049C40] Seib D.R., Corsini N.S., Ellwanger K. (2013). Loss of Dickkopf-1 restores neurogenesis in old age and counteracts cognitive decline. Cell Stem Cell.

[MJT049C41] Selkoe D.J. (2011). Alzheimer's disease. Cold Spring Harb. Perspect. Biol..

[MJT049C42] Selkoe D.J., Yamazaki T., Citron M. (1996). The role of APP processing and trafficking pathways in the formation of amyloid β-protein. Ann. NY Acad. Sci..

[MJT049C43] Shankar G.M., Walsh D.M. (2009). Alzheimer's disease: synaptic dysfunction and Aβ. Mol. Neurodegener..

[MJT049C44] Shen K., Cowan C.W. (2010). Guidance molecules in synapse formation and plasticity. Cold Spring Harb. Perspect. Biol..

[MJT049C45] Shimogori T., VanSant J., Paik E. (2004). Members of the Wnt, Fz, and Frp gene families expressed in postnatal mouse cerebral cortex. J. Comp. Neurol..

[MJT049C46] Terry R.D., Masliah E., Salmon D.P. (1991). Physical basis of cognitive alterations in Alzheimer's disease: synapse loss is the major correlate of cognitive impairment. Ann. Neurol..

[MJT049C47] Varela-Nallar L., Grabowski C.P., Alfaro I.E. (2009). Role of the Wnt receptor Frizzled-1 in presynaptic differentiation and function. Neural Dev..

[MJT049C48] Varela-Nallar L., Alfaro I.E., Serrano F.G. (2010). Wingless-type family member 5A (Wnt-5a) stimulates synaptic differentiation and function of glutamatergic synapses. Proc. Natl Acad. Sci. USA.

[MJT049C49] Varela-Nallar L., Parodi J., Farias G.G. (2012). Wnt-5a is a synaptogenic factor with neuroprotective properties against Aβ toxicity. Neurodegener. Dis..

[MJT049C50] Walsh D.M., Selkoe D.J. (2007). Aβ oligomers—a decade of discovery. J. Neurochem..

[MJT049C51] Zenzmaier C., Marksteiner J., Kiefer A. (2009). Dkk-3 is elevated in CSF and plasma of Alzheimer's disease patients. J. Neurochem..

